# On the impact of urban planning in contexts with limited enforcement of building and planning regulations: A study of the urban form of planned and unplanned informal settlements in Maputo, Mozambique

**DOI:** 10.1371/journal.pone.0292045

**Published:** 2023-09-28

**Authors:** Johan Mottelson

**Affiliations:** Institute of Architecture, Urbanism and Landscape, Royal Danish Academy, Copenhagen, Denmark; The City College of New York, UNITED STATES

## Abstract

**Background:**

More than one billion people live in informal settlements under precarious conditions. Urban planning is considered an important instrument to mitigate compromised living conditions in informal settlements. However, limited studies have investigated the long-term impact of urban planning in contexts with limited capacity to enforce building and planning regulations. The purpose of this study is to assess the long-term impact of urban planning on the development of sustainable urban form in contexts characterized by unregulated urban development.

**Methods:**

The study conducted geospatial surveys of three urban areas in Maputo, Mozambique covering adjacent planned and unplanned settlements that were established more than 40 years ago and subsequently developed with limited enforcement of building and planning regulations. High-resolution maps were produced and six urban form metrics were computed for the planned and unplanned areas respectively, providing the basis for quantitative and qualitative comparative analysis.

**Results:**

Although the study found signs of street encroachment and appropriation of the public space in the planned areas, the study found higher levels of built densities, higher proportions of public space, and higher average street widths in all planned areas compared to the respective neighboring unplanned areas. Furthermore, the statistical analysis consistently showed large effect sizes (Cohen’s d > 0.8) of urban planning on indicators of compact city development and access conditions.

**Conclusion:**

The results underscore that planning of street fabrics and plot layouts can enhance compact city development, improve transportation conditions, and increase the feasibility of investments in infrastructure in contexts with limited capacity to administer the urban growth.

## Introduction

Over one billion people reside in informal settlements characterized by lack of state recognition, contested rights to the land, and non-compliance with building and planning regulations [1]. Informal settlements present significant challenges to long-term sustainability as unregulated urban development and lack of state recognition can lead to unsafe construction, inadequate access to infrastructure, and insecure tenure [2–4]. More specifically, non-compliance with building regulations can lead to emergence of sub-standard housing conditions that increase exposure to disease and risk of building collapse [5, 6]. The extra-legal conditions typical of informal settlements and consequent lack of state recognition often deter authorities from investing in provision of basic infrastructure which can lead to unsanitary conditions in the public space [1]. As a result, informal settlements are associated with compromised living conditions leading to aggravate public health outcomes [7–9]. Ultimately, authorities can evict the residents of informal settlements without compensation because of the extra-legal construction and land occupation [10]. Consequently, the insecure tenure conditions can lead to risk of lost investments in housing and businesses, which can discourage long-term investment and undermine economic development [11]. Informal settlements pose particular challenges in sub-Saharan Africa, where the majority of the rapidly growing urban population resides in the informal housing sector [2].

Several aspects of the sub-standard living conditions commonly found in informal settlements are linked to spatial factors such as inadequate accessibility, high levels of building coverage, and limited public space [7, 12]. As mitigation of such urban deficiencies largely depends on urban planning and enforcement of building and planning regulations, spatial planning is regarded a crucial element in support of sustainable urban development [13–15]. Studies have suggested that unplanned urban development leads to duplicative costs of infrastructure, exacerbating issues related to inadequate provision of clean water, sanitation, and rainwater management systems in contexts characterized by rapid urban development and limited institutional capacity [16, 17]. As a minimal proportion of public space is critical for implementation of such infrastructure, street encroachment and resulting low proportions of public space common in informal settlements are crucial issues contributing to sub-standard living conditions [18]. Accordingly, the lack of enforcement of planning regulations can contribute to the development of unsustainable urban form and result in compromised living conditions in informal settlements.

A number of studies have emphasized suitable policy measures to support the development of sustainable urban form of informal settlements in sub-Saharan Africa. These strategies include early establishment of planned urban structures, in situ upgrading of established informal settlements, and state recognition of the land occupation [7, 10, 15, 19–20]. Establishment of planned urban structures during the early formation of settlements have been promoted to provide adequate space for infrastructure and vehicular access as well as to avoid subsequent costly structural readjustment of the settlements [15]. In situ incremental interventions, comprising street expansions and infrastructure improvements, along with wider recognition and land title provision initiatives have been promoted to improve living conditions in established informal settlements through higher level of tenure security and enhanced access to basic infrastructure [7, 19]. Simplification of the requirements of planning guidelines, land title acquisition procedures, and issuance of construction permits have been promoted to enable and advance legal urban development [10]. Provision of increased mandate for decision-making to the local level authorities have been promoted to address issues with limited institutional capacity to administer the urban development [10, 15]. Although many of these approaches have been examined in the scholarly literature, the long-term impact of implementation of planned urban structures during the formation of the settlements remains a under investigated. Notably, no research has investigated the long-term impact of urban planning during the early settlement formation on the development of sustainable urban form in contexts characterized by limited enforcement of building and planning regulations.

In the context of Maputo, Mozambique, previous research reported four distinct categories of informal settlements in Maputo: (1) formally planned areas recognized by the state, (2) unplanned areas lacking state recognition, (3) planned areas lacking state recognition, (4) unplanned areas recognized by the state [21]. Notably, the planned settlements that lack state recognition encompass areas that have been planned by local government, international organizations, or local private sector planners. However, these areas lack formal recognition by the municipality as planned zones. Consequently, construction within such areas remains extra-legal, as issuance of construction permits requires alignment with approved urban plans that guide the urban development. These conditions have led to the emergence of informal settlements with planned structural layouts that have evolved under limited state governance. The purpose of the present study is to investigate the long-term impact of urban planning in contexts where planning regulation is enforced to a limited degree through comparison between adjacent unplanned and planned informal settlements. More specifically, the study addresses the following research question: Does urban planning have a long-term impact on the development of sustainable urban form in contexts characterized by limited enforcement of building and planning regulations?

To answer the question, the study analyzed the urban form characteristics of adjacent planned and unplanned settlements in Maputo which were established more than 40 years ago and subsequently developed with limited enforcement of building and planning regulations. These areas were surveyed using UAVs (drones) and GPS trackers for production of high-resolution geo-referenced orthophotos which were manually delineated and used for computation of urban form metrics. The study employed an analysis of quantitative urban form metrics extracted from the geospatial surveys along with a qualitative investigation of the respective planned and unplanned urban fabrics based on visual inspection of the manually delineated maps.

Although the analysis does not provide insights into the socioeconomic and cultural dimensions of the development of the neighborhoods, it highlights the spatial outcome of these processes [3]. Documenting the long-term impact of urban planning on the development of urban form in contexts with limited institutional capacity has the potential to improve the basis for decision-making in contexts characterized by informal urban development. Accordingly, the relevance of the study extends beyond Mozambique and the African continent as proliferation of unplanned informal settlements occurs in rapidly urbanizing areas with limited institutional capacity in a range of low-income countries globally.

## Materials and methods

The study relies on (1) high-resolution geospatial surveys of case study areas covering adjacent planned and unplanned informal settlements; (2) manual delineation of buildings, streets, and trees; (3) computation of urban form metrics of the planned and unplanned informal settlements; (4) quantitative analysis the urban form metrics and qualitative analysis of the manually delineated maps.

### Study setting

**Fig 1:**
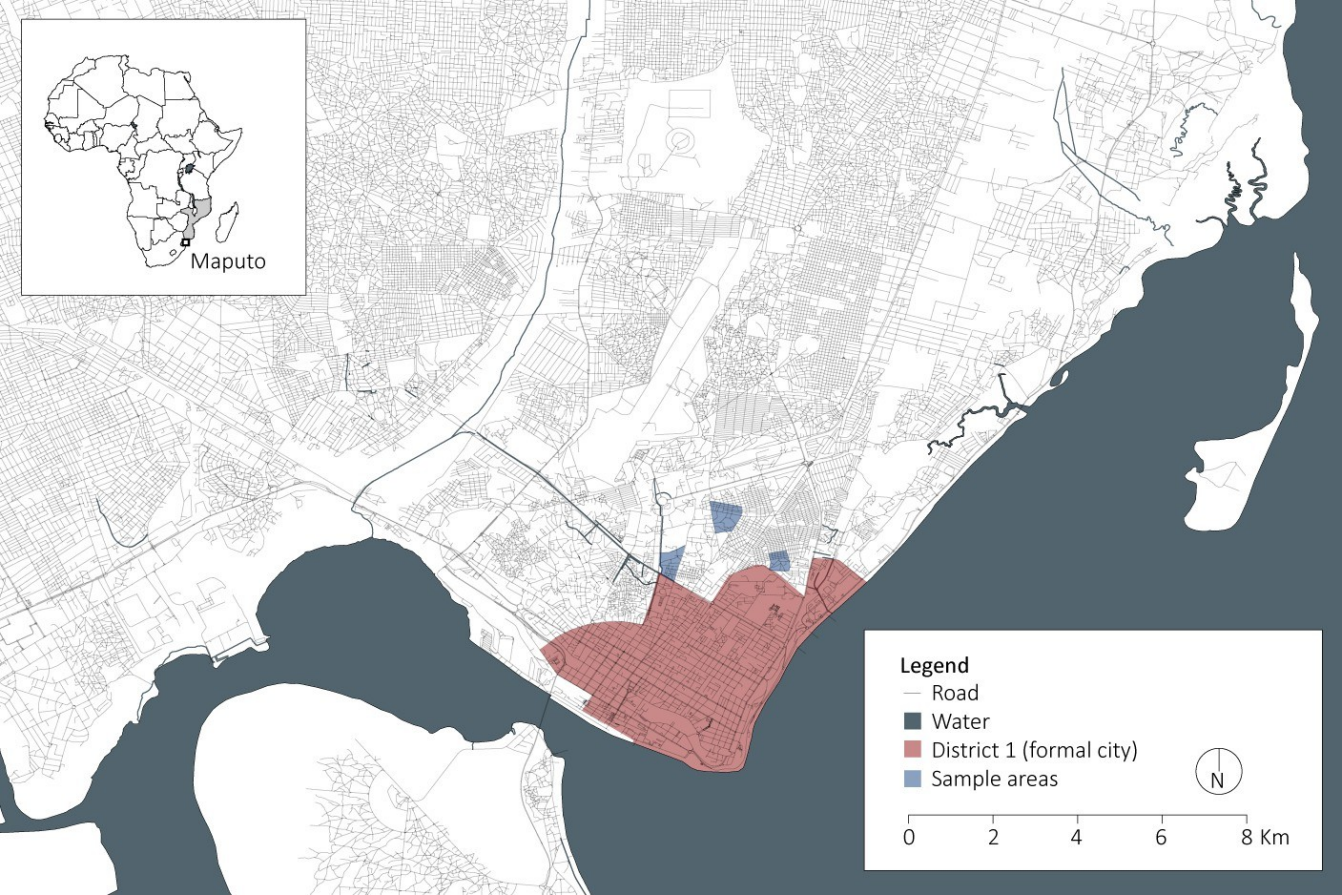
Overview of Maputo. Source: OpenStreetMap contributors. (2015) Planet dump [Data file from 11.11.2021]. Retrieved from https://planet.openstreetmap.org. Graphics prepared by Johan Mottelson.

Mozambique is a low-income country located in southeast Africa and Maputo is the capital as well as social, economic, and cultural center of the nation. Since Independence, most of the urban growth of Maputo has been accommodated through proliferation of informal settlements and the majority of the population in the city resides in the informal housing sector [22]. The study was conducted in three centrally located informal peri-urban areas in Maputo, Mozambique located in KaMaxakeni (District 3) covering parts of the Maxaquene (A, B, and D) and Polana Caniço A neighborhoods (see Fig 1). These areas were selected as they comprise neighborhoods with similar socioeconomic profiles and presence of adjacent planned and unplanned informal settlements established around the same time.

The sample areas were largely established in the period after Mozambique gained independence from Portugal during which the population of Maputo grew rapidly. However, although no academic sources exist on the planned area in Maxaquene A, it was established prior to independence as can be observed in historical aerial photos. The planned areas in Maxaquene D and Polana Caniço A included in the study were established as a part of the Urbanization Project of Maxaquene and Polana Caniço (1977–1979) developed by the National Directorate of Housing and funded by the United Nations [23]. As a part of the project, residents of the neighborhoods were relocated in order to implement structural readjustment resulting in the planned urban fabrics seen today [24]. The urban fabrics of the planned areas comprise orthogonal street grids, blocks measuring approximately 50x30 meters, and plots measuring approximately 15x10 meters. The adjacent unplanned examined areas are characterized by an organic urban form (i.e., no planned structure of the street layout) as well as diverse sizes and geometries of both plots and blocks. Although parts of the neighborhoods are planned, they lack state recognition as planned areas [21]. Construction in these areas is thus extra-legal, as construction permits cannot be formally issued in areas that lack approved urban plans to guide the urban development. Both the planned and unplanned examined areas are thus informal settlements as both are characterized by extra-legal occupation of the land and extra-legal construction (see [25, 26]). There are no records of planning interventions in the neighborhoods since the implementation of the Urbanization Project of Maxaquene and Polana Caniço (1977–1979). The respective planned and unplanned sample areas are mostly flat and characterized by similar topographical conditions, and there are no significant other geographic differences across the planned and unplanned sample areas. However, it is possible that parts of the vegetation were removed to establish the street and plot structures in the planned sample areas during implementation. Accordingly, all of the planned and unplanned sample areas are characterized by limited enforcement of building and planning regulations, extra-legal construction and land occupation, similar socioeconomic profiles, and limited differences in terms of geographic factors.

### Geospatial surveys

The geospatial surveys used in the study were authorized by local authorities at district (*distrito*) and neighborhood (*bairro*) levels using a standardized form for conducting fieldwork obtained from the Faculty of Architecture and Physical Planning, Eduardo Mondlane University (*Faculdade de Arquitectura e Planeamento Físico*, *Universidade Eduardo Mondlane*) to obtain authorization to capture aerial photographs of the sample areas. The detailed geospatial surveys of all planned and unplanned sample areas were conducted in June and July 2019, using a drone (UAV) for systematic collection of high-resolution photometric data. Utilizing Pix4D photogrammetry software, the aerial photos (photometric data) were processed to generate geo-referenced orthophotos, Digital Surface Models (DSM), and digital 3D models of each of the examined areas (see [27]). The demarcation of the boundaries of the examined settlements was based on major roads, which were excluded from the dataset to mitigate data bias. A smartphone equipped with the SW maps application was employed using GPS features to track the boundaries of the public space within each settlement (see [12]). These data (GPS traces, orthophotos and DSMs) were subsequently imported into QGIS, where they were placed and automatically scaled and exported in DXF format. The DXF files were imported into AutoCAD, where distinct layers were created for buildings, trees, blocks, and the street network.

### Manual delineation of key features of the urban environment

**Fig 2:**
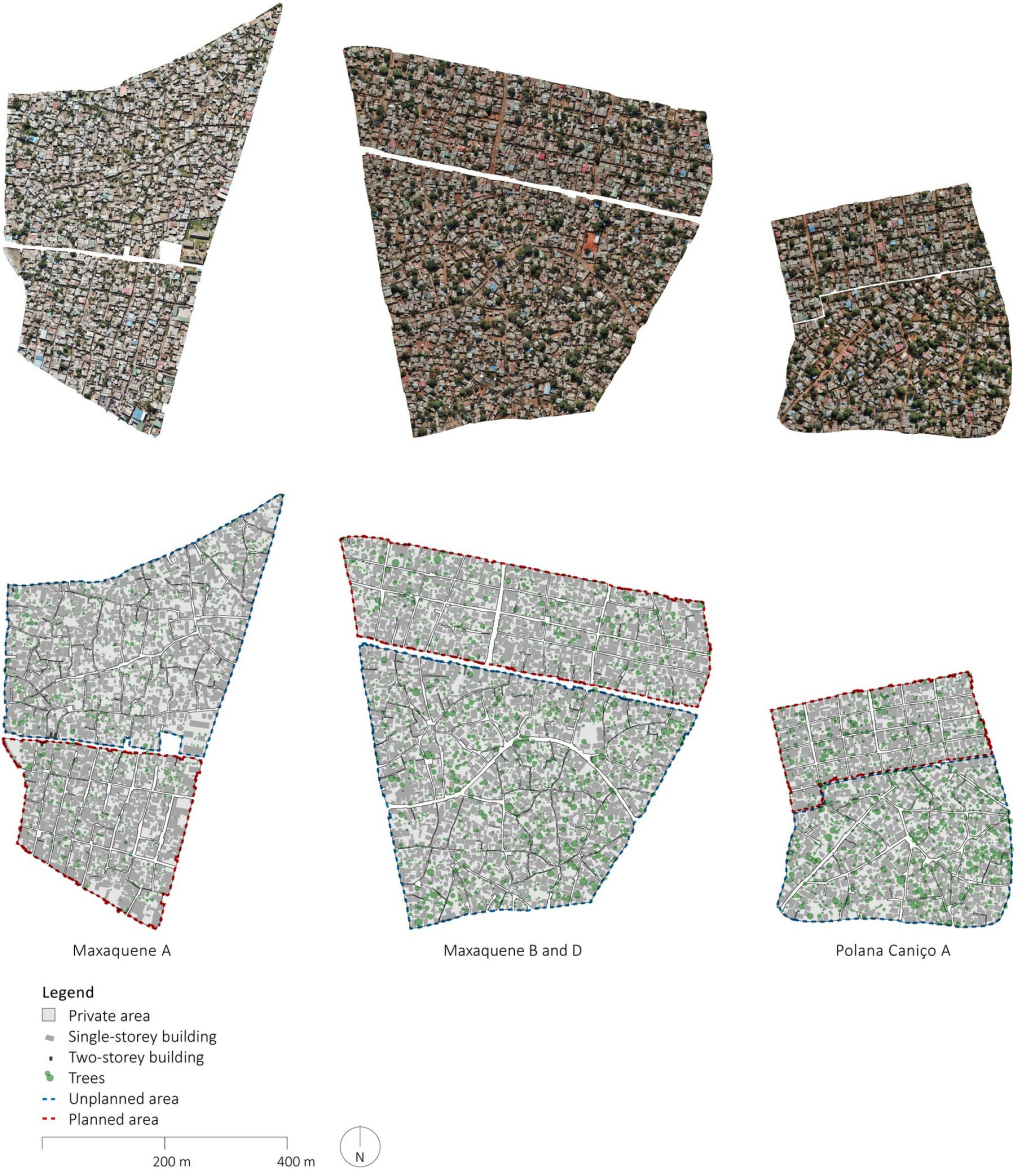
Overview of outputs of geospatial surveys. The orthophotos were generated using UAVs for systematic collection of photometric data and Pix4D for data processing. The maps were created by manually annotating the orthophotos using QGIS and AutoCAD. Source: Johan Mottelson.

**Fig 3:**
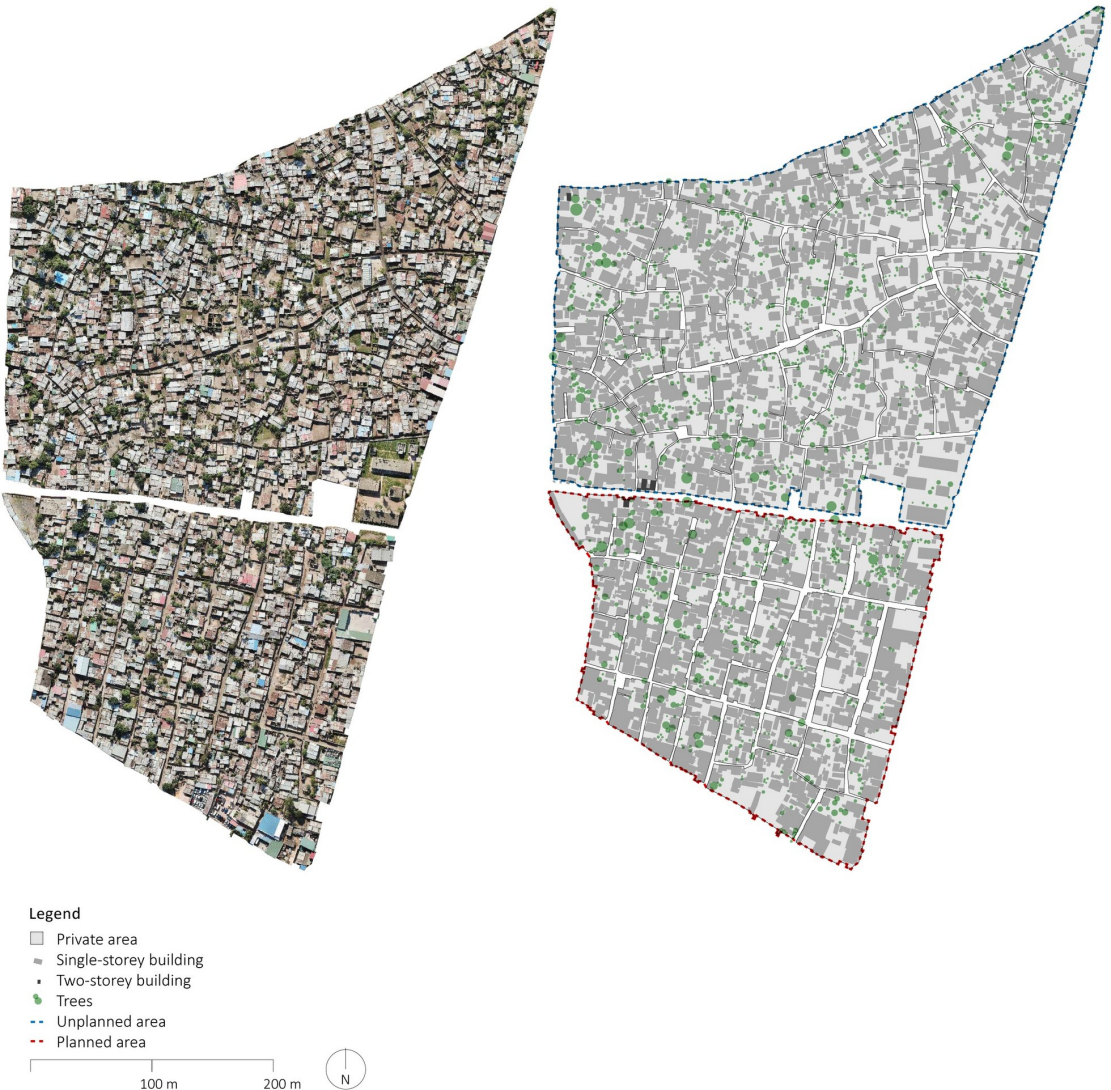
Orthophoto and manually delineated map of Maxaquene A. The orthophoto was generated using UAVs for systematic collection of photometric data and Pix4D for data processing. The map was created by manually annotating the orthophoto using QGIS and AutoCAD. Source: Johan Mottelson.

**Fig 4:**
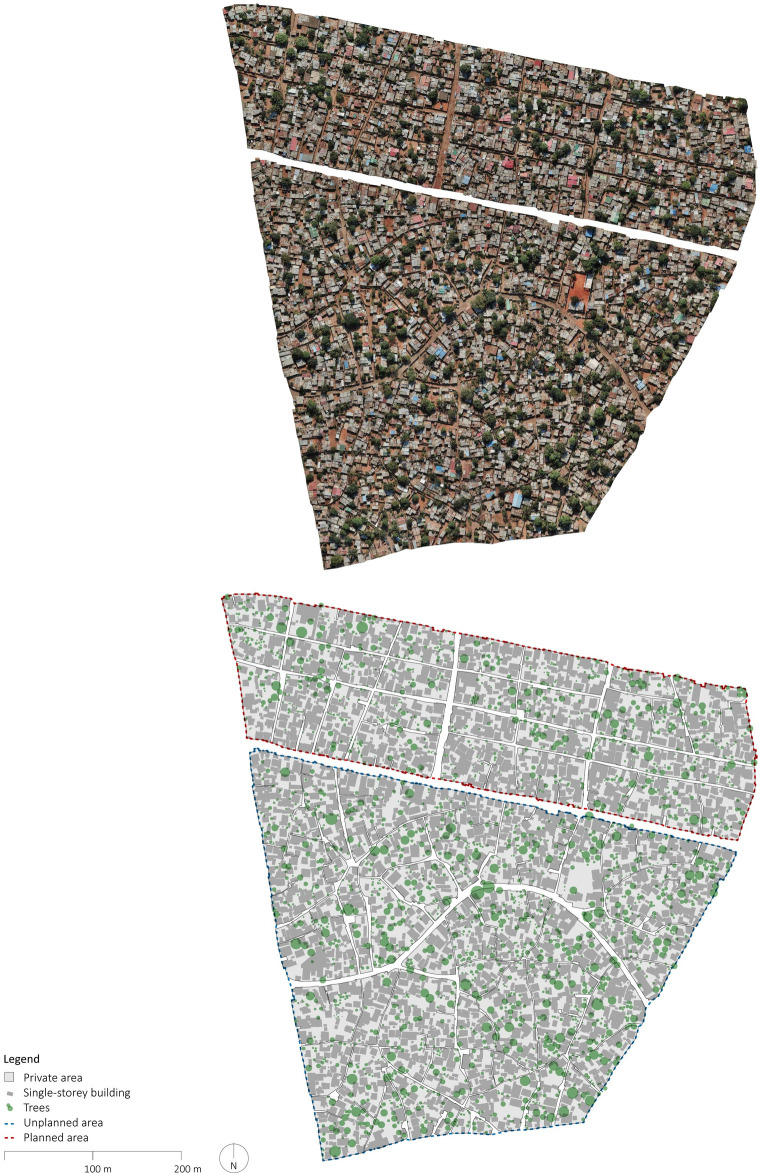
Orthophoto and manually delineated map of Maxaquene B and D. The orthophoto was generated using UAVs for systematic collection of photometric data and Pix4D for data processing. The map was created by manually annotating the orthophoto using QGIS and AutoCAD. Source: Johan Mottelson.

**Fig 5:**
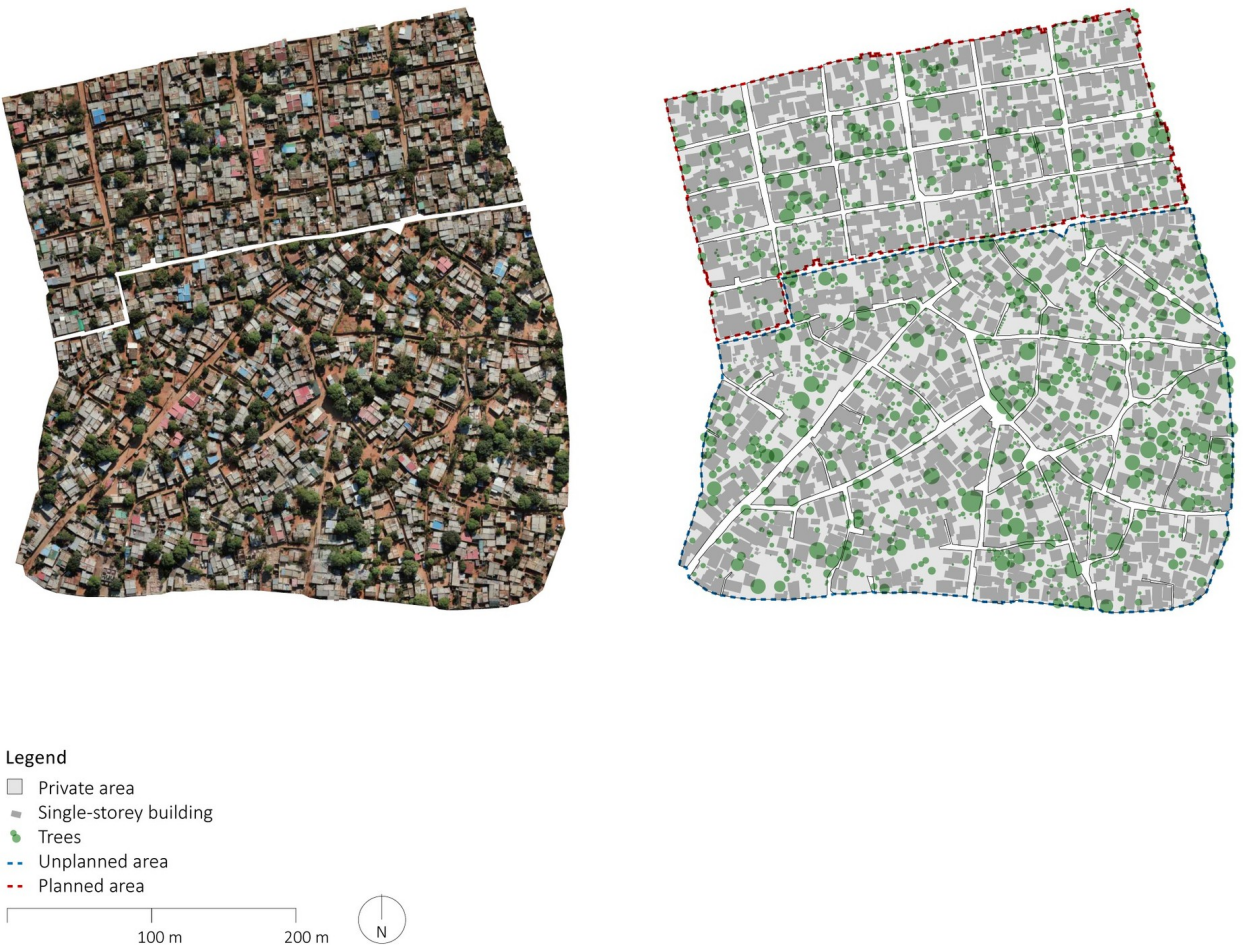
Orthophoto and manually delineated map of Polana Caniço A. The orthophoto was generated using UAVs for systematic collection of photometric data and Pix4D for data processing. The map was created by manually annotating the orthophoto using QGIS and AutoCAD. Source: Johan Mottelson.

**Fig 6:**
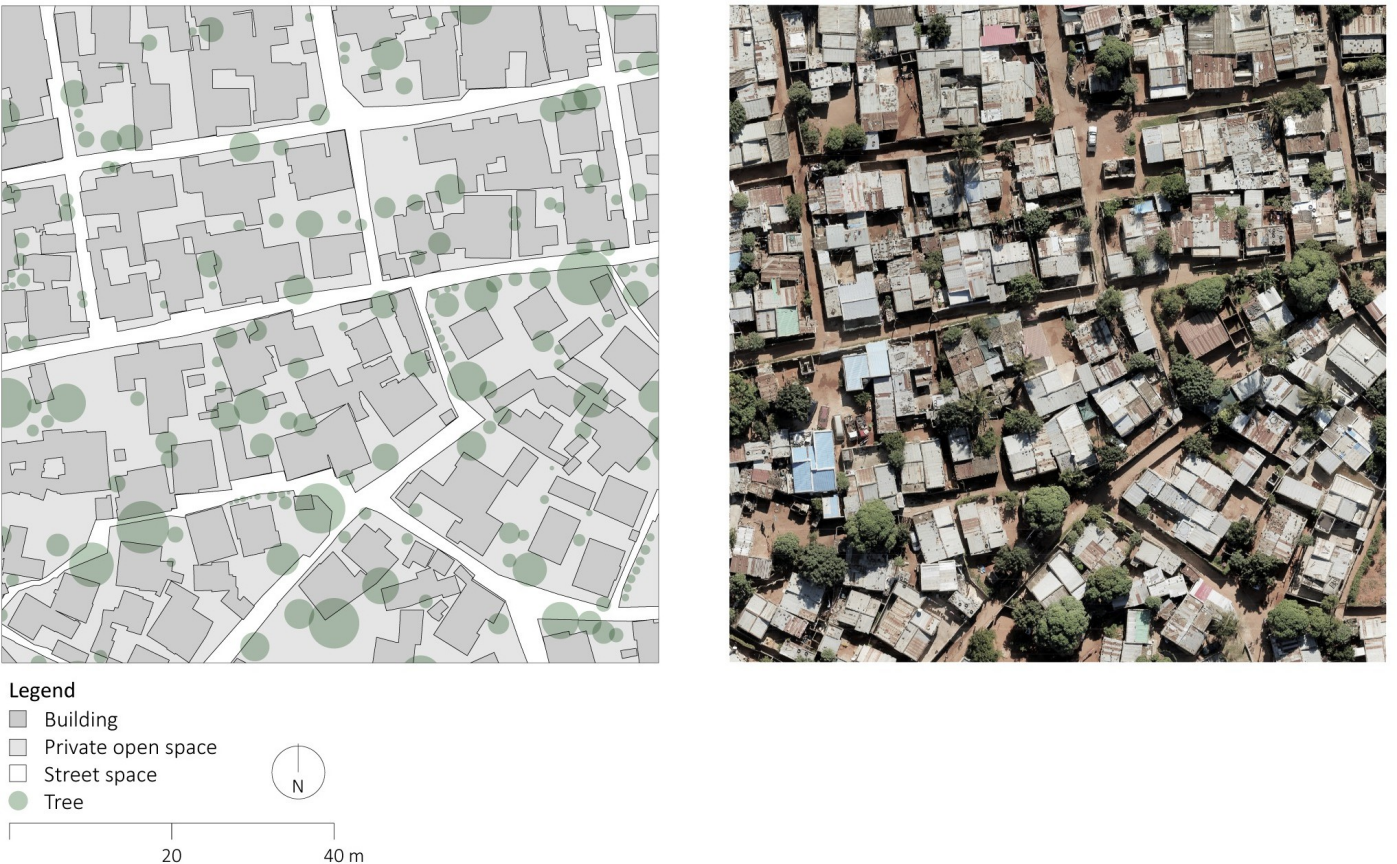
Detailed section of orthophoto and manually delineated map of Polana Caniço A. The orthophoto was generated using UAVs for systematic collection of photometric data and Pix4D for data processing. The map was created by manually annotating the orthophoto using QGIS and AutoCAD. Source: Johan Mottelson.

Buildings, streets, and trees were manually delineated based on the GPS traces, detailed orthophotos, DSMs, and 3d model. More specifically, the boundaries of all buildings were traced on the basis of the orthophoto. Each floor of multi-story buildings was delineated and annotated based on building heights and identification of each floor based on the DSMs and the 3d models. The street network and the public space were identified based on the GPS traces and orthophotos enabling delineation of the boundaries of all blocks. Trees were delineated with circles corresponding to the canopy observed in the orthophotos. The outputs, including orthophotos and manually delineated maps of each of the planned and unplanned sample areas are presented in Figs 2–6.

### Computation of urban form metrics and analysis

The manually delineated maps enabled automatic extraction of the total area of buildings (building footprints and area of all floors), private space (blocks), and tree canopy cover. In addition, the number of blocks along with the total length of both the street network and the building perimeters were automatically computed and extracted from AutoCAD. The total public space area was calculated by subtracting the total private space area from the total area of each sample area. These data were used to compute six indicators of urban form, including Floor Area Ratio (FAR), Public Space Ratio (PSR), Average Block Size (ABS), Surface Area to Volume ratio (SAV), Average Street Width (ASW), and Urban Tree canopy Cover (UTC).

These indicators were selected because of their respective importance in relation to scholarly discussions on urban form and sustainable transformation of informal settlements. Urban density measures such as FAR and SAV where included because compact city development is widely acknowledged for its beneficial effects on economic development, enhanced mobility, and reduced costs of infrastructure and service delivery [28]. This is particularly important in the context of sub-Saharan Africa, where urban development is largely characterized by inadequate infrastructure provision and compromised mobility. Accordingly, indicators used to measure compact city development is of importance for assessing if urban planning contributes to development of sustainable urban form in contexts with limited institutional capacity to enforce planning regulations. Secondly, inadequate provision of basic infrastructure such as water, sewage and drainages is a key issue related to informal urban development in sub-Saharan Africa [1]. Such infrastructure is typically arranged in the street space, and low proportion of public space compromises the feasibility of provision of said infrastructure. Indicators used to measure the public space such as PSR and ASW provide insights into the feasibility of improving the infrastructure provision. Decreased mobility and inadequate access conditions are common features of informal urban development, because of low proportion of public space, narrow lanes and constrained pedestrian of permeability of the urban fabrics [12]. Large block sizes and dead-end-streets comprise indicators of such conditions. To measure conditions for pedestrian mobility, block sizes across the sample areas were surveyed. Finally, urban tree canopy cover is linked to local micro-climate [29], rainwater infiltration [30], and biodiversity. Notably, lack of vegetation has been linked to increased exposure to dangerous heat in dense informal settlements in sub-Saharan Africa [31]. On this basis, UTC was included in the study to measure potential differences in vegetation density across the planned and unplanned sample areas.

- **The following section presents a concise overview of the six urban form indicators utilized in this study:** Floor Area Ratio (FAR) is a metric used to measure the intensity of land use and building density. FAR is defined as the ratio of the total building floor area to the total land area [32]. FAR can be calculated at plot level or at neighborhood level. In this study it was calculated based on the total floor space and the total private space to alleviate data bias resulting from variations in public space across sample areas.
- Public Space Ratio (PSR) is a metric used to measure the proportion of the public space relative to the total sample area. The public space is defined as areas of a neighborhood which the public has legal access to, such as streets, sidewalks, and parks [33]. PSR is defined as the ratio of the public space area to the total land area [34].
- Average Block Size (ABS) is a metric highlighting the scale of the urban fabric. A block consists of private space surrounded by public space [32]. ABS is defined as the total private area divided by the number of blocks.
- Surface Area to Volume ratio (SAV) is a metric highlighting the compactness of the built environment. SAV is defined as the total building surface area divided by the volume of all buildings [35]. The calculation of surface area involved summing the perimeter length of all buildings, multiplied by the height of each floor, and adding the total building footprint area multiplied by two. The volume was determined by summing the area of each floor, multiplied by the corresponding floor height.
- Average Street Width (ASW) is a metric highlighting the access conditions. ASW is defined as the total area of the public space divided by the total length of the street network [36].
- Urban Tree canopy Cover (UTC) is a metric highlighting the density of trees. UTC is defined as the total ground area covered by the crowns of trees divided by the total sample area [37, 38].

**Fig 7:**
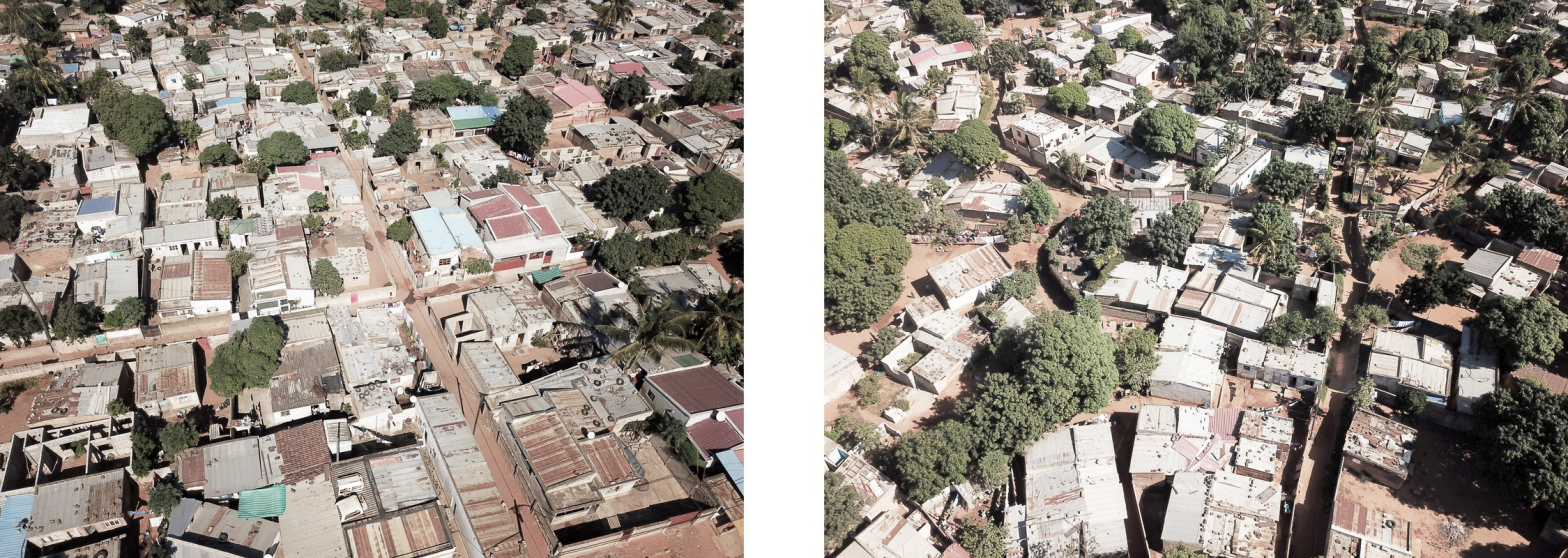
Aerial photographs of planned and unplanned sample areas in Polana Caniço A. Source: Johan Mottelson.

**Fig 8:**
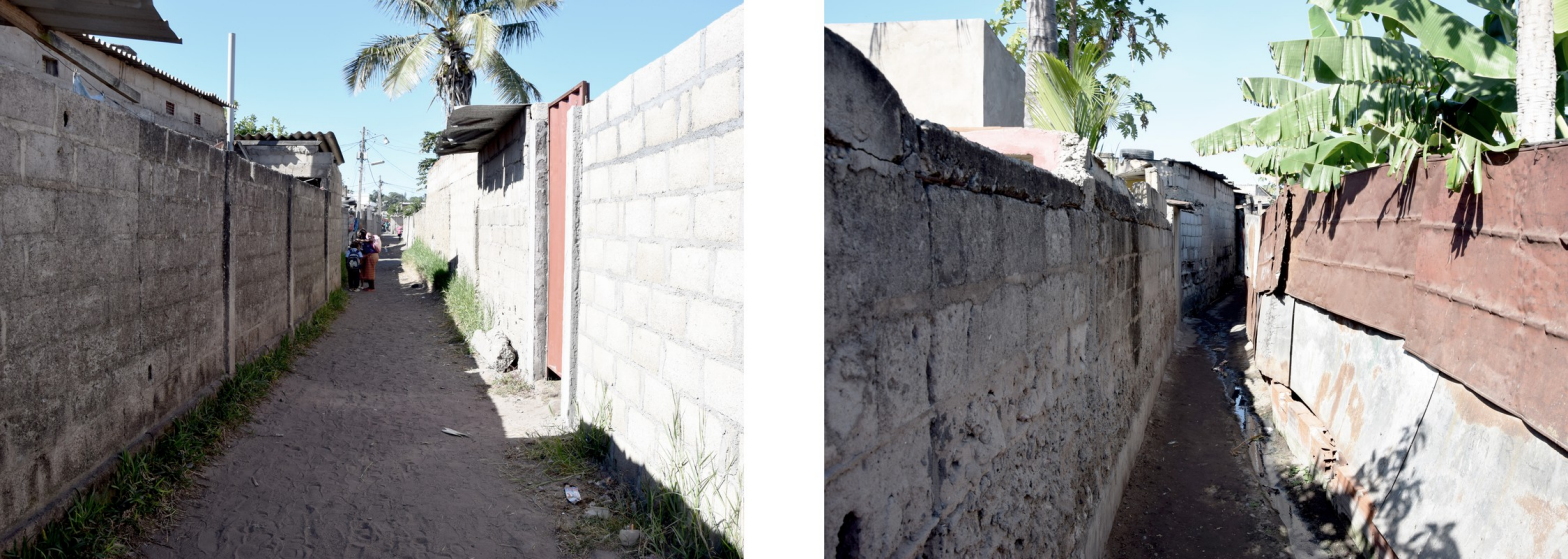
Street photographs of planned and unplanned sample areas in Maxaquene A. Source: Johan Mottelson.

Each of these six metrics were computed for each planned and unplanned sample area providing the basis for the quantitative analysis of the urban form of the adjacent planned and unplanned informal settlements. Mean values and standard deviation across all planned and all unplanned sample area were computed for each indicator. Effect sizes (Cohen’s d) were computed to provide standardized representations of the magnitude of differences on selected indicators between planned and unplanned areas. Effect sizes were interpreted based on established thresholds: values around 0.2 indicate a small effect size, around 0.5 indicate a moderate effect size, and around 0.8 or higher indicate a large effect size [39]. Photos of planned and unplanned sample areas recorded during the field work are presented in Figs 7 and 8.

### Qualitative analysis

The qualitative analysis relied on visual inspection of the manually delineated maps [40]. This approach encompassed an examination of the spatial organization of the street layout and the arrangement of buildings supplementing the quantitative analysis. In particular, variations in street widths within planned areas were regarded as potential evidence of gradual encroachment upon the street space. Furthermore, deviations in the planned urban fabric geometry were interpreted as indicative of a broader appropriation of the public space. Additionally, the alignment between streets and buildings was considered an indication of a more optimized utilization of space. The presence of elongated dead-end pathways was regarded as a sign of reduced pedestrian permeability. These distinctive features were discerned through qualitative examination of the maps and accompanying photographic material (Figs 2–8).

## Results

The six indicators of urban form computed for each settlement under examination are summarized in Table 1. The data show higher levels of built density (FAR), more compact urban form (SAV), higher levels of public space (PSR), and larger average street width (ASW) in all three planned urban areas compared to the respective three adjacent unplanned urban areas. On average the planned areas have a 14% higher FAR, 27% higher PSR, 20% higher ASW, and 10% higher SAV compared to the unplanned areas. The average block size (ABS) was 23% larger in unplanned areas. However, in one case (Maxaquene B and D) the ABS was almost identical in the adjacent planned and unplanned areas. The urban tree canopy cover (UTC) was on average 24% higher in unplanned areas. However, in one case (Maxaquene A) the UTC was slightly higher in the planned area.

**Table 1:** Urban form data.

	Planned sample areas					
	FAR	PSR	UTC	ASW (m)	SAV	ABS (m^2^)
Maxaquene A	0.5149	0.1072	0.0593	2.99	1.9995	2,365.07
Maxaquene B-D	0.4638	0.1003	0.0971	3.16	1.8815	3,289.20
Polana Caniço A	0.4836	0.1116	0.1299	2.98	2.0532	1,597.66
Mean	0.4874	0.1064	0.0954	3.05	1.9781	2,417.31
Standard deviation	0.0258	0.0057	0.0353	0.10	0.0878	846.98
	Unplanned sample areas					
	FAR	PSR	UTC	ASW	SAV	ABS
Maxaquene A	0.4657	0.0714	0.0521	1.98	1.7828	2,887.41
Maxaquene B-D	0.4297	0.0837	0.1334	2.41	1.7666	3,244.93
Polana Caniço A	0.3775	0.0922	0.1909	2.88	1.7918	3,249.47
Mean	0.4243	0.0824	0.1255	2.43	1.7804	3,127.27
Standard deviation	0.0443	0.0105	0.0697	0.45	0.0128	207.74
Effect size (Cohen’s d)	1.7	2.8	0.5	1.9	3.2	1.2

The statistical analysis documents large effect sizes (Cohen’s d > 0.8) of urban planning on five out of six indicators of urban form. The largest effect size was observed on SAV (Cohen’s d = 3.2), indicative of a consistently more compact built environment and more optimized land use efficiency of construction across the planned urban areas. Similarly, a large effect size on FAR was observed (Cohen’s d = 1.7), indicative of a consistently denser built environment across the planned sample areas. Notably, large effect sizes were also observed concerning PSR (Cohen’s d = 2.8) and ASW (Cohen’s d = 1.9), indicative of the consistently improved access conditions and larger proportion of public space found across all planed areas. A large effect size was furthermore observed concerning AWB (Cohen’s d = 1.2), indicative of a consistently higher level of pedestrian porosity across the planned sample areas. A moderate effect size was observed regarding UTC, indicative of lower vegetation density in planned urban areas.

The qualitative analysis of the urban fabrics highlights variations in street widths within planned areas indicative of gradual encroachment upon the street space (see Fig 7). Furthermore, inconsistencies in the planned urban fabric geometry were identified in Maxaquene A and to a lesser extent in Maxaquene B, indicative of appropriation of entire streets (see Figs 3 and 4). Additionally, streets and buildings were found to be more aligned in the planned areas compared to the unplanned areas, indicative of a more optimized utilization of space in the planned areas. The presence of elongated dead-end pathways was identified in all unplanned areas, indicative of reduced pedestrian permeability, consistent with the larger block sizes observed in the unplanned areas.

## Discussion

The study analyzed the urban form of adjacent planned and unplanned settlements in Maputo, Mozambique that were established more than 40 years ago and subsequently development with limited enforcement of building and planning regulations. The purpose of the study was to document the spatial impact of urban planning in contexts characterized by limited enforcement of building and planning regulations. The findings consistently reveal notable qualitative and quantitative differences across the planned and unplanned areas that have critical implications for infrastructure provision, accessibility, and livelihood. Notably, all of the examined planned areas are characterized by higher levels of built density, more compact urban form, higher proportions of public space, and higher average street widths compared to the respective adjacent unplanned areas. Notably, the statistical analysis consistently showed large effect sizes of urban planning on indicators of compact city development (FAR and SAV) as well as accessibility and pedestrian porosity (PSR, ASW, and ABS), while showing a moderate effect size on vegetation density (UTC). The qualitative analysis furthermore, documented variations in street widths and inconsistencies of the street network geometry, indicative of street encroachment and appropriation of the public space.

### Public space

The study found that the proportion of public space and the average street widths were higher across all three planned areas compared to all three unplanned areas. More specifically, the proportion of public space was on average 27% higher in the planned areas while the average street width was 20% higher. In addition, the average block size was generally larger and dead-end pathways more common in unplanned areas. Notably, large effect sizes (Cohen’s d = 1.2–2.8) of urban planning were consistently found across indicators of the public space, including proportion (PSR), access conditions (ASW), and pedestrian porosity (ABS). The differences notably imply that the planned areas feature near-universal car access facilitated adequate street widths, while the unplanned areas exhibit limited car access attributable to constrained road networks. According to UN-Habitat, street space should cover at least 30% of the land area in a neighborhood to support adequate mobility systems [41]. However, streets space covered only 8% of the unplanned areas and 11% of the planned areas on average. Other studies suggest that limited public space in informal settlements leads to duplicative costs of infrastructure provision [42]. As the study found higher proportions of public space in the planned areas, the study indicate that spatial planning can enhance feasibility of infrastructure provision in informal settlements in spite of limited enforcement of building and planning regulations (see [42–44]). Regardless of the limited enforcement of building and planning regulations, the planned urban areas notably require less in situ interventions to mitigate the issues related to unregulated urban development (see [7, 45]).

### Compact city development

The study consistently found more compact urban form (e.g., higher levels of FAR and SAV) in the planned settlements compared to the respective neighboring unplanned settlements. Furthermore, large effect sizes (Cohen’s d 1.7–3.2) were observed for FAR and SAV. This suggests that the planned areas are characterized by fewer, more compact and larger buildings or that the buildings are more conjoined while the unplanned areas are characterized by smaller and more scattered buildings. In addition, the qualitative assessment of the maps underscores that buildings in the planned areas are more aligned with the streets. These findings indicate that the planned areas are characterized by more optimized land use efficiency. Compact city development can accommodate a higher population density, that result in shorter commuting distances, reducing time and resource consumption on transportation [43, 44]. Higher dwelling densities also enable infrastructure provision at a lower cost per unit because of reduced distance between each dwelling [43, 44]. On this background, compact city development is promoted by international agencies due to the beneficial effects of higher urban densities on sustainability, resilience, mobility, and economic growth as well as decreased costs of service delivery and infrastructure provision [43, 44]. These concerns are particularly important in the context of sub-Saharan Africa, because of inadequate infrastructure provision, limited investment capacity for increasing infrastructure provision, as well as constrained mobility [46]. As the urban form metrics computed in the study consistently document more compact urban form in the planned areas (14% higher FAR and 10% higher SAV on average along with large effect sizes on these indicators), urban planning can enhance compact city development and thereby support development of sustainable urban form in contexts characterized by limited enforcement of building and planning regulation.

### Tree cover

The study found a higher average urban tree canopy cover in the unplanned areas along with a moderate effect of urban planning on UTC (Cohen’s d = 0.5). Lack of trees can lead to increasing surface urban heat island effect compromising local micro-climate [29] and links between lack of vegetation and increased exposure to dangerous heat in informal settlements in Africa have been documented [31]. Furthermore, rainwater infiltration in urban soils is estimated to be higher in areas with lawns with trees [30]. Accordingly, higher levels of urban tree canopy cover will likely contribute to mitigation of issues with storm water management. In addition, trees constitute an important volume of the urban biomass. Accordingly, the lower levels of urban tree canopy cover found in the planned areas may compromise urban micro climate, rainwater management, and biodiversity. Previous longitudinal research of tree canopy cover in unplanned informal settlements in Maputo underscore that vegetation density generally is declining [47], which can have potential adverse effects on urban micro climate, rainwater infiltration and bio-diversity. Accordingly, declining vegetation density is a broader tendency across informal settlements in the context of the study and likely endemic within planned areas.

### Cross segment variations

While the study found higher levels of built density, higher proportions of public space, and larger average street widths in all planned sample areas compared to the adjacent unplanned areas, the study also found some variations in these data across the three planned areas. The qualitative assessment of the urban fabric highlighted that the urban fabric of the planned area in Maxaquene A is characterized by irregularities which are likely the result of residents appropriating entire streets. Furthermore, small variations in street widths and shifts in extensions of plots and buildings into the street space observed at local level across all examined planned areas underpin that small-scale appropriation of the public space through street encroachment is a common practice. As critical differences in such practices are both observed qualitatively and quantitatively across the sample areas, this is indicative of different local administrative practices in the planned informal settlements. More specifically, street encroachment and wider appropriation of the public space is accepted to a varying degree, underscoring the importance of local administrations in informal settlements in administering the urban development.

### Potential mechanisms accounting for the findings and future research

A number of questions arises on the background of the analysis for future research to investigate. What explains the higher levels of built density and more compact urban form in the planned areas? Why are the planned areas characterized by lower tree canopy cover? Are the higher levels of built density observed in the planned areas the outcome of smaller plots and higher density of households? Do households with higher socioeconomic status who can afford to construct larger houses seek to live in planned areas because of better access conditions for cars? Is the more compact urban form in the planned areas an outcome of the simpler plot geometry which is more optimized for efficient configuration of buildings? However, as the study does not document the sociocultural dynamics that account for the differences observed between the planned and unplanned areas [3], it is unable to directly address these inquiries. Nonetheless, this final section of the discussion presents several mechanisms that may explain the findings. It is important to emphasize that these remain speculative and cannot be conclusively determined based on the results of the study alone; hence, further research is needed to explore their validity. As such, this final section identifies and discusses relevant domains for future research in order to address questions arising from the present study.

The formation of street-systems and plot boundaries are essential for the long-term urban development as they typically remain unchanged through successive generations of society [48]. Accordingly, the higher proportions of public space and higher average street widths found in the planned sample areas are likely linked to urban planning during the early formation of the settlements. In addition, the simple uniform urban fabric of the planned sample areas may be easier for local authorities to manage as it is likely easier to identify street encroachment in the planned areas compared to the unplanned areas. As streets and buildings occupy a higher proportion of the land in the planned areas, less open space is vacant for vegetation. Accordingly, higher levels of built density and higher proportions of public space in the planned areas may account for the lower urban tree canopy cover. However, as noted in the context description, vegetation may have been removed as a part of the implementation of the respective planned urban structures which may have contributed to the lower tree canopy cover in the planned sample areas.

Informal housing in Maputo is characterized by simple rectangular building layouts optimized for standardized construction materials [49]. The simple rectangular plot geometry in the planned areas may be more optimized for land use efficiency through configuration of simple rectangular buildings compared to the irregular plot geometries in the unplanned areas. In addition, it is possible that plot sizes are smaller in in the planned areas, resulting in higher household densities. Thereby, smaller plots in planned areas may partially account for the higher levels of built-up densities in the planned sample areas due to higher densities of dwellings and thereby also increased collective investment capacity for densification of the built environment (see [3, 50]). Furthermore, there may be socioeconomic differences between planned and unplanned areas as residents with more economic resources likely prefer to reside in areas with better accessibility of cars. As research has documented links between other types of amenities and socioeconomic status of residents [51], it is likely that car accessibility increases land values and thereby may contribute to an upward trajectory in socioeconomic status through successive land transactions processes over the course of time. This may contribute to higher investment capacity in planned areas enabling construction of larger and more compact dwellings resulting in higher built-up densities. Accordingly, smaller plot sizes and higher socioeconomic status may thereby account for the higher levels of built density in planned areas. Notably, studies from other parts of the world have documented higher levels of urban tree canopy cover in suburban areas characterized by higher socioeconomic status [38], while the reverse dynamic may characterize the context of the present study. Namely, that higher socioeconomic status of residents of peri-urban Maputo may be associated with lower vegetation density. Accordingly, future research on the socioeconomic characteristics of planned and unplanned informal settlements hold potential to enhance to our understanding of the cultural values and amenity preferences in the context of peri-urban cities in sub-Saharan Africa critical for future sustainable urban development.

## Conclusions

The study investigated the impact of urban planning in contexts with limited capacity to enforce building and planning regulation based on analysis of planned and unplanned sample areas in peri-urban Maputo, Mozambique established more than 40 years ago. The study found higher levels of built density, more compact urban form, higher proportions of public space, and higher average street widths in all of the examined planned areas compared to the adjacent respective unplanned areas. In addition, the study showed large effect sizes of urban planning on indicators compact city development as well as pedestrian and vehicular accessibility. The study thus demonstrates that a basic level of urban planning can have a long-term impact on the urban form of informal settlements despite limited enforcement of urban regulation. The study highlights that urban planning can enhance compact city development and improve access conditions in informal settlements. This may increase the cost-efficiency of investments in infrastructure and increase the impact of such investments. Accordingly, the study suggests that a basic level of urban planning can support sustainable urban development in rapidly urbanizing contexts with limited institutional capacity.

## Supporting information

S1 File Impact of planning data. Manually delineated features of the urban environments of all sample areas used in the study. The high-resolution orthophotos are available via openaerialmap.org.
(DWG)

S2 File Inclusivity-in-global-research-questionnaire. Mandatory form about ethical aspects of conducting research in a global context.
(DOCX)
